# Recent Advances in Shiga Toxin-Producing *Escherichia coli* Research in Latin America

**DOI:** 10.3390/microorganisms6040100

**Published:** 2018-09-28

**Authors:** Alfredo G. Torres, Maria M. Amaral, Leticia Bentancor, Lucia Galli, Jorge Goldstein, Alejandra Krüger, Maricarmen Rojas-Lopez

**Affiliations:** 1 Department of Microbiology and Immunology, Sealy Institute for Vaccine Sciences, University of Texas Medical Branch, Galveston, TX 77555, USA; 2 Laboratorio de Fisiopatogenia, Departamento de Fisiología, Instituto de Fisiología y Biofísica Bernardo Houssay, Facultad de Medicina, Universidad de Buenos Aires, Buenos Aires C1121ABG, Argentina; mmamaral74@gmail.com; 3 Laboratory of Genetic Engineering and Molecular Biology, Institute of Basic and Applied Microbiology, National University of Quilmes, Bernal, Buenos Aires 1876, Argentina; lbentan@unq.edu.ar; 4 Instituto de Genética Veterinaria Ing. Fernando N. Dulout (UNLP-CONICET, La Plata), Facultad de Ciencias Veterinarias, La Plata 1900, Argentina; lgalli@igevet.gob.ar; 5 Consejo Nacional de Investigaciones Científicas y Técnicas (CONICET), Instituto de Fisiología y Biofísica Houssay, Facultad de Medicina, Universidad de Buenos Aires, Buenos Aires C1121ABG, Argentina; jogol@fmed.uba.ar; 6 Centro de Investigación Veterinaria de Tandil (CONICET-CIC-UNCPBA), Facultad de Ciencias Veterinarias, Tandil 7000, Argentina; akruger@vet.unicen.edu.ar; 7 Department of Medicine, Division of Infectious Diseases, Massachusetts General Hospital, Harvard Medical School, Boston, MA 02115, USA; maricarmen.rojaslopez@gmail.com

**Keywords:** Shiga toxin, STEC, Shiga toxin-producing *E. coli*, enterohemorrhagic *E. coli*, hemolytic uremic syndrome, diarrhea, vaccines, therapeutics, phages

## Abstract

Pathogenic *Escherichia coli* are known to be a common cause of diarrheal disease and a frequently occurring bacterial infection in children and adults in Latin America. Despite the effort to combat diarrheal infections, the south of the American continent remains a hot spot for infections and sequelae associated with the acquisition of one category of pathogenic *E. coli*, the Shiga toxin-producing *E. coli* (STEC). This review will focus on an overview of the prevalence of different STEC serotypes in human, animals and food products, focusing on recent reports from Latin America outlining the recent research progress achieved in this region to combat disease and endemicity in affected countries and to improve understanding on emerging serotypes and their virulence factors. Furthermore, this review will highlight the progress done in vaccine development and treatment and will also discuss the effort of the Latin American investigators to respond to the thread of STEC infections by establishing a multidisciplinary network of experts that are addressing STEC-associated animal, human and environmental health issues, while trying to reduce human disease. Regardless of the significant scientific contributions to understand and combat STEC infections worldwide, many significant challenges still exist and this review has focus in the Latin American efforts as an example of what can be accomplished when multiple groups have a common goal.

## 1. Introduction

Shiga toxin-producing *Escherichia coli* (STEC) is a pleiotropic group of isolates which include several serogroups and serotypes, and which have in common the presence in their genomes of a bacteriophage encoding the Shiga-toxin genes [1]. Some of these serotypes, particularly the predominant O157:H7, have become important food- and water-borne pathogens that can cause a range of symptoms in humans, from diarrhea and hemorrhagic colitis to hemolytic-uremic syndrome (HUS) [2]. STEC isolates are found in animals, cattle being the most important reservoir of STEC strains, and the source of contamination of food products and water [3]. Multiple serogroups are found in cattle and other animals, but only certain STEC serogroups (commonly known as enterohemorrhagic *E. coli* [EHEC], due to their capacity to cause disease in humans), such as O26, O45, O103, O111, O121, O145, and O157, pose a large economic burden to food producers because of massive product recalls due to the significant risk to human health [4]. A study published in 2014 evaluating the global incidence of human STEC infections and deaths estimated that STEC causes more than 2.8 million acute illnesses annually, leading to 3890 cases of HUS, 270 cases of end-stage renal disease and 230 deaths [5]. Public health efforts in many countries have resulted in a reduction in the number of outbreaks and even if an outbreak occurs, the sequelae in affected individuals has also been ameliorated.

**Figure 1:**
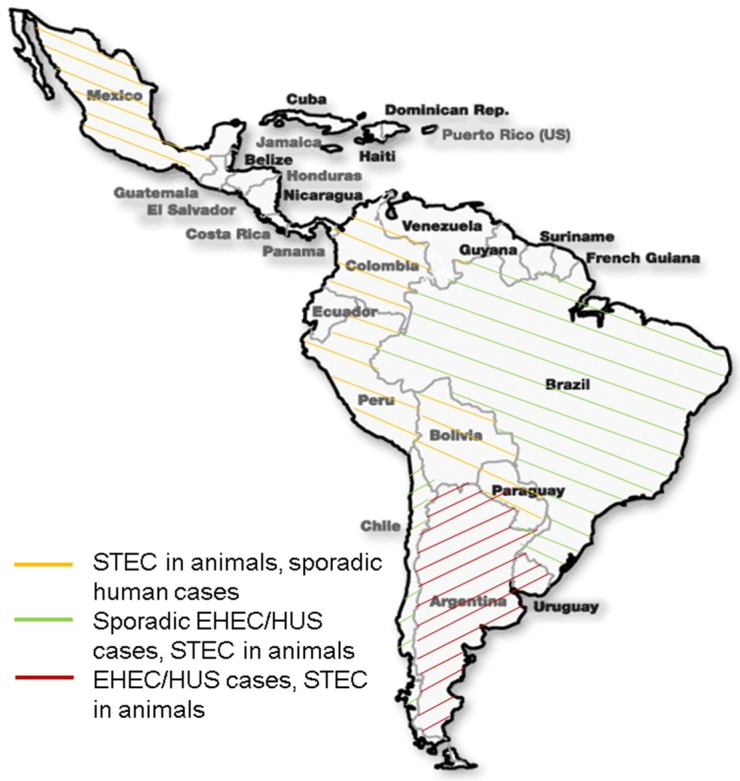
Geographic distribution of STEC/EHEC isolates and cases in Latin America. The map present the predicted distribution of STEC isolates in Latin American countries based on different epidemiological studies [6,7]. Different STEC serogroups are present in animals, mainly cattle, throughout the region, but the majority of EHEC/HUS human cases are localized at the southern part of the continent.

In contrast for Latin America, STEC infections remain endemic and contribute to the burden of acute diarrheal syndrome in this region (Figure 1) [6,7]. It has been estimated that STEC infections account for approximately 2% of total cases of acute diarrhea and 20%–30% of bloody diarrhea [8]. Like in many parts of the world, EHEC O157:H7 remains the main serotype associated with human infections, with a significant number of HUS cases mainly localized in the southern countries of the continent (Argentina, Chile, Uruguay) [8]. In Argentina, post-diarrheal HUS is endemic. From 2000–2010, approximately 500 HUS cases were reported annually. The incidence has been estimated to be in a range of 7.8%–17% per 100K children less than five years of age and the lethality ranged between 2%–5% (the incidence is 10-fold higher than any other industrialized country). Interestingly, the genetic homogeneity of STEC O157 serogroups detected in human and bovine strains are similar; however, as suggested by some groups, a zoonotic cycle exist with differential expression of virulence and colonization factors, which could explain the persistent phenotype in different environments and reservoirs [9].

Coordinated efforts by Ministries of Health and health professionals in many of these countries have tried to improve isolation, diagnosis, treatment and food safety. Despite these efforts, it was reported in Argentina in 2016 that around 400 HUS cases continue to be reported annually, with an incidence of 8.4 cases/100K children less than five years of age and a lethality ranging from 2%–5% [10]. Because STEC/EHEC remains a significant health problem in this region of the world, the number of Latin American investigators working in different aspects of STEC/EHEC pathogenesis, epidemiology, treatment, diagnosis, public health and safety, have increase significantly and as an example, this review article will highlight recent efforts from the Latin American research community to advance basic and translational research to further understand the virulence traits of the diverse STEC isolates, particularly focusing on the pathobiology of the Shiga toxins, the phages encoding those genes and further advance the knowledge regarding environmental distribution of isolates in animals and food products. Finally, a summary of the organized efforts as a research community to attack the problem of STEC/EHEC infections using the principles of One Health is discussed. Due to the nature of this review, seminal work by groups outside Latin America was not included or referenced; however, the authors want to recommend these excellent reviews and books for further reading [1,7,11,12,13,14,15,16,17].

## 2. Discovery of STEC Serotypes and Novel Virulence Factors in Isolates from Humans and Animals

The emergence of new STEC strains is a clear evidence of the dynamic genome that pathogenic *E. coli* isolates possess and their ability to transfer or acquire important virulence factors. In recent years, several Latin American groups have evaluated the appearance of emergent strains, their relationship to natural reservoirs and diversity of their virulence factors and importance in the pathogenesis of STECs. In South America, mainly in Argentina, the most common serotype is O157:H7 (Figure 1), and the molecular characterization of different isolates is important to distinguish their distribution and their genetic diversity among different geographical regions. A molecular epidemiology study of O157 strains isolated from different regions of Argentina showed that all these strains harbored *rfbO157*, *fliCH7*, *eae*, and *ehxA* genes, and a *stx2a*/*stx2c* predominant genotype in both human and bovine strains [18,19]. It was shown that the strains displayed diverse genetic fingerprint patterns, but two common patterns found were already included in the Argentine Database of *E. coli* O157. It was also found that the hypervirulent STEC O157:H7 clade 8 is prevalent, circulating among these isolates, in contrast to other countries were clade 7 is prevalent [9]. Further, a relation between incidence of HUS and the O157 present in cattle was proposed [18,19].

Another study using pediatric isolates from the same country, found higher prevalence of STEC in HUS than in diarrheic patients, with O157:H7 as the most prevalent, followed by O145:NM. Most of the O157:H7 were antibiotic sensitive, carrying *stx2*, *eae*, *fliC H7*, *ehxA*, *iha, efa*, *toxB*, *lpfA1-3* and *lpfA2-2* [20]. There were other *eae*-negative isolates which belonged to a variety of serotypes, but overall this study showed that STEC of diverse serotypes and genotypes are circulating in a same region. The characterization of O157 isolates can be mapped to each specific region and their unique clades or linages; however, this does not happen for non-O157 isolates. In Argentina, the most prevalent non- O157 STEC serogroups are O145, O121, O26 and O174, being the latest an important emerging pathogen [21]. Serotypes O174:H21 and O174:H28 isolated from animals, food, and human showed that all the strains possess the *afaC* gene and are negative for *eae* and *toxB* genes. Further analysis of other important virulence factors indicate that O174:H21 carry *lpfO113* (*lpf2-1*), *iha*, *ehxA*, *saa*, *subA*, and *stx2c* subtype; while O174:H28 possess *iha* and *subA*, *lpfO113* (*lpf2-1*), *ehxA*, *saa* and *stx2a* subtype [22]. Although STEC O174 are not included in any international standard regulations, surveillance has been recommended at least for Argentina and neighboring countries.

Further, STEC O157:H7 has also been a prevalent strain in Brazil. However, it was until recently that the first human case associated with foodborne disease and caused by *E. coli* O157:NM, a strain previously found exclusively in calves, was reported [23]. This strain was isolated from a 13-year-old boy, suffering from intense abdominal pain and severe diarrhea, mesenteric adenitis, and then bloody diarrhea. The infection occurred after eating a tomato and cheese salad a week before the onset of the symptoms [23]. Another example of the genome plasticity of STEC strains and their capacity to evolve is represented by the atypical enteropathogenic *E. coli* (aEPEC) strains which have been isolated from stool samples of Brazilian diarrheic patients, isolates which carry O157-plasmid genes (*ehxA* and/or *espP*) [24]. These aEPEC serotypes included O26:H11, O76:H- and O145:H- (positive for *ehxA* and *espP* genes) and O85:H4 (positive for *espP*). The presence of O157 genes in these aEPECs suggest that they might have derived from STEC strains that have lost the *stx* genes, because it has previously been reported that O26:H11 and O145:H- are well-recognized STEC serotypes [24].

Other studies investigating the prevalence of STEC strains as a public health risk have screened and isolated STEC strains from different sources, including synanthropic animals. STEC isolates were identified (*stx1* and/or *stx2* positive but *eae* negative) in the order Artiodactyla in zoo animals from Chile, isolating STEC for the first time from Thomson’s gazelle (*Eudorcas thomsonii*) [25]. Other studies have detected STEC strains in stool samples from asymptomatic children and domestic animals (cattle, a guinea pig, and a chicken) from households in a semirural community in Ecuador; however, STEC symptomatic disease in humans seems to be rare in this country [26]. Further, strains positive for the *stx2* gene have been isolated from captive birds in Brazil [27]. Another study has led to the isolation of STEC strains from rodents (*Rattus spp*.) in Argentina. These STEC strains belonged to the O108:H11 serotype and carry the *eae*, *stx1*, *stx2*, *rfb*O157 and *subA* genes [28]. The enterohemolytic phenotype, which has been associated with diarrheal disease in humans, was observed in all these STEC isolates, although it was not possible to establish a connection between isolated strains and affected population.

To evaluate virulence phenotypes of new isolated strains, it is important to perform an analysis of the virulence factors located within and outside pathogenicity islands (PAI), which are important for unique STEC virulent phenotypes. The correlation and the distribution of 20 important virulence genes encoded within PAIs, in those called O-islands (OI-), different than the Locus of Enterocyte Effacement (LEE), were evaluated for their involvement in STEC pathogenesis. The screening included clinical samples as well as vegetables or meat and cattle isolates, and all of them were Stx (Stx1 or Stx2) positive [29]. The virulence genes tested were effectors encoded on OI-36, OI-57, OI-71 or OI-122, with special emphasis on the genes from OI-122 (6 ORFs which were rearranged in 3 modules), which have been associated to outbreaks and severe disease along with the LEE. The results showed different profiles for each PAI encoded in the STEC isolates; however, the most prevalent gene in LEE-positive and LEE-negative strains was Z4321, a gene encoded in OI-122 and displaying homology to PagC from *Salmonella enterica* serovar Typhimurium. Further, approximately 20% of the strains presented none of the 20 genes investigated as markers. Finally, several profiles for the PAIs were associated to these isolates and might be predictable of human risk [29].

More recently, a new PAI, called Locus of Adhesion and Autoaggregation (LAA), was characterized in STEC LEE-negative strains. This PAI is an 86-kb island composed of 80 genes, organized into four modules and the first three modules were present in several LEE-negative clinical isolates [30]. LAA encodes several virulence factors, including the iron-regulated gene A homologue adhesin (Iha) and an autotransporter adhesin known as antigen 43 (Ag43). This locus also encodes a heat-resistant agglutinin (Hra) protein, non-previously described. This hemagglutinin was renamed Hemagglutinin from Shiga toxin-producing *E. coli* (Hes) and was shown to be functional and able to confer colonization-associated phenotypes to a non-adherent *E. coli* strain, agglutination of sheep erythrocytes, autoaggregation, biofilm formation and adherence to epithelial cells in an aggregative pattern [30]. More importantly, it was shown that Hes, Ag43 and NmpC (a heat resistance protein found in LAA) proteins are reactive to sera from HUS patients. This supports the idea that LAA proteins might be important for the pathogenesis of LEE-negative strains. It has been suggested that in the absence of LEE, the LAA island might confer an alternative adhesion mechanism, aggregative (or “semi-localized”), during intestinal colonization in humans, conferred by Iha, Hes, and Ag43 [30]. LAA is present among STEC LEE-negative STEC strains of clinical relevance, such as some isolated from Hemorrhagic colitis or HUS cases, from serotypes O91:H21, O174:H21 and some O113:H21 [30], and serogroups from animal origin: O91, O174, O113, O171, O178, O130, and others [31]. Overall, the presence of this PAI has been associated with *stx2*-positive strains and belonging to phylogroup Bl.

## 3. Novel Insights about Shiga Toxins and the Immune Responses

It is known that once the Shiga toxins enter the bloodstream, they might trigger HUS, a described triad of events which includes thrombocytopenia, microangiopathic hemolytic anemia, acute renal failure [32], and/or central nervous system (CNS) alterations [4,8]. Latin American researchers have extensively contributed to the discovery of novel insights into mechanisms involved in STEC-mediated damage to organs and; therefore; at the study of beneficial drugs that can neutralize the deleterious effects by Stx or Subtilase (SubAB) toxins, as described below by target organ affected.

Blood cells: it is known that Soluble CD40-ligand (sCD40L), released from platelets, binds to CD40 on target cells and triggers an inflammatory response [33] or produce oxidative stress and reactive oxygen species (ROS) in various cellular types, including endothelial cells or monocytes. Abrey Recalde et al. [34], investigated the effects of Stx2 and oxidative stress on renal microvasculature, platelet adhesion, and sCD40L release to identify a novel mechanism contributing to thrombotic microangiopathy. The in vitro experiments and blood assessment obtained from HUS patients revealed a new pathway of platelet-monocyte interaction mediated by sCD40L. Further, oxidative stress data suggested an additional progression of endothelial dysfunction during Stx2-associated HUS by activated platelet sCD40L release. It was suggested that antioxidant treatments may be useful to reduce platelet activation and thrombus formation, improving renal microcirculation and kidney function. Overall the results indicated that quantification of plasma sCD40L could be used as a marker of microvascular dysfunction/or platelet activation leading to thrombotic risk in HUS patients, since plasma levels of sCD40L were found elevated, similarly to other pathological conditions such as cardiovascular diseases, diabetes or HIV infection [35,36,37].

Another study has shown that Stx binds to polymorphonuclear neutrophils (PMN), delaying apoptosis, inducing ROS production, increasing expression of activation markers, such as CD11b and CD66b, and triggering the formation of extracellular traps (NETs). In addition, a correlation between monocytosis and HUS severity was observed in Stx-HUS patients, together with changes in the expression of chemokine receptors on these cells. Further, monocytes suffer activation during Stx-HUS, contributing to renal endothelial glomerular damage [38]. These results have leaded to the conclusion that not only Stx had direct cytotoxic effects on endothelial or leucocyte cells, but also are able to elicit inflammatory responses during HUS pathogenicity.

Kidney: A novel in vitro model of human renal tubule epithelial cells was developed based on an in vitro 3D model, to study the mechanisms of tubular repair and regeneration after injury. The relevance of this study is the new concept indicating that Stx2 is able to inhibit the mechanisms of de-differentiation and re-differentiation, affecting the regenerative capacity of human renal tubular epithelial cells [39]. Seyahian et al. [40], have evaluated the systemic effects of SubAB toxin in rats. In this regard, it has been observed that in intoxicated animals, among other events, accumulation of peritoneal ascites, and histological alterations in heart, colon, liver and kidneys occur. In the kidney, microalbuminuria was associated with the observed alteration of glomerular filtration function, including the transformation of the villous podocytes and the effacement of their foot processes. The alterations were suggestive of the disruption of the filtration barrier, and the decrease of megalin expression in proximal tubules, as a factor in tubular protein reabsorption failure. Such events occurred under the influence of inflammatory mediators, like TGF-β, indicating that inflammation mechanisms participate actively in the development of renal failure. Another study by Amaral et al. [41], has previously demonstrated that in vitro exposure of Stx2 or SubAB toxins decreases the viability of human glomerular endothelial cells (HGEC) and human proximal tubule epithelial cells (HK-2) by inducing apoptosis. Subsequent studies evaluated whether ouabain (OUA) was able to protect cells from toxin effect. They found that nanomolar concentrations of OUA protected HGEC viability from Stx2 and SubAB treatment and only protected HK-2 cells viability from Stx2 intoxication. These protective effects were associated with the prevention of apoptosis by increasing cell proliferation and suggested that OUA might have therapeutic use, preventing HUS-renal injury [42]. Further, the effects of Stx2 and SubAB on HGEC and HK-2 cells have been investigated by co-culturing both cells, resembling a human renal proximal tubule model of water absorption and cytotoxicity in the presence of Stx2 and SubAB. Both toxins inhibited the net absorptive water transport across HGEC and HK-2 monolayers and this effect was independent of cell viability [43]. It was postulated that the toxins could cause direct alterations in the mechanisms involved in water transport across endothelial and/or epithelial monolayers, but lack of inhibitory effects on water movement argues that the protective effect is due to close-proximity of the endothelium/epithelium. In addition, it was shown that both toxins inhibited cell viability in HGEC and HK-2 monolayers but not in the constituted bilayer [43]. These original investigations contributed to the understanding of pathophysiological mechanisms that alter renal functions by both toxins.

Brain: A multi-centric, observational, retrospective and cross-sectional study was recently conducted by the Argentine National Epidemiological Surveillance System of HUS concluded that Central Nervous System (CNS) involvement was the main predictor of death in patients suffering from STEC-HUS [16]. In view of the clinical relevance of neurological disorder derived from HUS, neurobiological research in Latin America has been conducted in the past few years and novel information has emerged from this research. One new concept was the finding that Stx2 has a direct action in the brain; as local administration of this toxin on rats produced brain damage in neurons, astrocytes, oligodendrocytes and endothelial cells [44]. Because the Gb3 receptor was immuno-detected in rodent brain neurons, Stx2 may act through Gb3 neuronal receptor [45]. Further, the neurological damage found in HUS patients was extrapolated to a murine model of sub-lethal Stx intoxication [46]. Using this model, it was observed that Stx2 breaks the blood-brain-barrier (BBB) and damages cells that modulate motor functions [47]. Further, reactive astrocytes and neurodegeneration were the most important outcomes which correlated with motor dysfunction. Under these conditions, Stx2 was immuno-detected inside neurons that upregulated the Gb3 receptor. Contrary to this, the administration of dexamethasone, an anti-inflammatory drug, protected mice challenged against 2 DL_100_ from death, and partially reversed the observed deleterious effects of the toxin [47]. Further studies, using integrative physiological, behavioral and ultrastructural studies, were performed to evaluate cerebellar function effects caused by Stx2 treatment. Sub-lethal Stx2 concentrations altered the BBB permeability in the cerebellum of mice. This event allowed the toxin to penetrate the cerebellar parenchyma, leading to damage of Purkinje cells [48]. This model can be used to study the cerebellar syndrome observed in HUS patients and to provide clues in how to prevent neurologic damage. Finally, another study has evaluated angiotensin-(1–7), a brain protectant of the renin-angiotensin system and a reducer of microglia activation and pro-inflammatory cytokine production, against the deleterious effects of Stx2 in the brain. Stx2 was administered locally in the anterior hypothalamus of rats’ brains and it was observed that Ang-(1–7) protects mainly neurons and oligodendrocytes, preventing axon demyelination and partially astrocyte reactivity, and suggesting that this peptide might exert an anti-inflammatory action [49]. Overall, these studies have revealed that inflammatory responses appear to be a decisive event in the progression of HUS-derived encephalopathy. A recent report confirmed that methylprednisolone, a corticoid anti-inflammatory drug, normalized a patient from acute encephalopathy [50]. The cumulative basic and clinical reports revealed that anti-inflammatory therapy should be seriously considered as a treatment for acute HUS-derived encephalopathies.

## 4. Progress in Phage Therapy and *Stx* Phage Biology

It has been already emphasized that within Latin America, Argentina has the highest incidence of HUS worldwide and the most frequent serotype is O157:H7, representing 60% of cases [6,7]. Approximately 20% to 30% of patients with HUS suffer serious sequelae, such as chronic renal failure, requiring dialysis or transplantation [51]. In addition, damage to the central nervous system can develop, with a poor prognosis if symptoms of severe neurological damage occur at the onset of the disease [16]. Pharmacological interventions in HUS therapy have been empirical and limited, the use of anticoagulants and anti-thrombotic agents has not fully worked [13,52], and antibiotics are contraindicated due to inefficiency, or even counterproductive, since they would produce massive release of toxin by inducing the excision of the bacteriophage [4,8]. Although progress has been made in the knowledge about the pathogenic mechanisms of HUS, specific therapies are not yet available. Therefore, new therapeutic approaches are necessary to control HUS and the role of bacteriophage has been evaluated.

Bacteriophage 933W is a temperate bacteriophage inserted into STEC strains and the gene coding for Shiga toxin is found in the genome of this lambda-like bacteriophage [53]. During STEC infection, the excision and replication of the bacteriophage occurs and Shiga toxin (Stx) is expressed and released. Subsequently, free bacteriophages can infect other susceptible bacteria in the gut, exacerbating bacteriophage replication and Stx expression [53]. Interestingly, Tyler et al. [54], have published that STEC mutant strains in the prophage cleavage mechanism do not induce renal disease.

The ability of mammalian cells to express Stx2 in vitro has also been investigated [55]. The study utilized bioinformatics analysis of the promoter sequences of *stx2* and found that the promoters of the A and B toxin subunits had a 97%–99% probability of being recognized by the eukaryotic cell machinery. Using recombinant plasmids containing the gene coding for GFP (Green Fluorescent Protein) under the control of eukaryotic promoters on the subunit A (pr1) or subunit B (pr7), it was shown that GFP expression was observed in cells transfected with these constructs. Similarly, a plasmid containing the *stx2* gene under its own promoter (pStx2) resulted in cytotoxicity of transfected cells and the phenotype was eliminated when pre-incubated with anti-Stx2 antibodies. Overall, this study demonstrated that bacteriophage 933W can enter macrophages in vitro [55].

This study was validated in vivo [56], by inoculating Balb/c mice with pStx2 and the animals died with typical signs of Stx2 poisoning, including kidney damage, neutrophilia, brain damage, and with detection by immunofluorescence of Stx2 in the brain. Further, mice immunized against the toxin B subunit, survived the pStx2 inoculation. These results demonstrate the ability to express Stx2 in vivo by maintaining a toxic activity equivalent to the toxin produced by the bacteria during infection.

Based on these studies and demonstration of the capacity of the lambdoid bacteriophage to be internalized by mammalian cells, a new line of research emerged looking at the bacteriophage as a potential therapeutic target, with the aim of blocking the infection of STEC in the gut and subsequent entry of Stx into host cells [53]. As a result, chitosan [57] and cationic peptides [58], have been founded to have anti-bacteriophage capacity. Chitosan is a linear polysaccharide composed of randomly distributed chains of β-(1–4) D-glucosamine (deacetylated units) and *N*-acetyl-d-glucosamine (acetylated unit). It presents a large number of commercial and biomedical applications and it is approved by the FDA (Food and Drug Administration) for human use. Ly-Chatain et al. [59], reported anti-bacteriophage activity of this compound against bacteriophage c2, which infects *Lactococcus* strains, and also had an effect on bacteriophage MS2, which infects *E. coli* strains.

Anti-bacteriophage activity of chitosan on the bacteriophage 933W was observed under in vitro and in vivo conditions [57], inhibiting the ability of the bacteriophage to infect *E. coli* strains. This compound was further evaluated as a potential anti-bacteriophage agent in vivo by testing its ability to inhibit the bacteriophage infective properties on susceptible strains. If this inhibition occurs in the intestine, it would prevent the replication of the bacteriophage and the expression of Stx. As a result, a decrease in deaths was observed in mice infected with STEC who received chitosan after infection [57].

Alternatively, anti-bacteriophage activity of antimicrobial peptides have been tested and it was demonstrated that some of them present a significant anti-bacteriophage in vitro activity [58]. Altogether, these studies have supported the role of bacteriophage 933W in HUS development, and support the idea that looking for anti-bacteriophages agents can be an effective alternative therapeutic approach against STEC infections.

## 5. Current Advancements in Control of STEC in Food Products and Food Safety

Food safety programs try to delineate the guidelines to generate food products that do not cause health problems to the population. Nevertheless, the hazards should always be determined by a risk assessment to help businesses identifying and managing those potential risks and, when necessary, apply the correct improvement actions.

Some general studies focused their attention in determining the STEC prevalence and/or frequency in certain food matrices. However, other studies try to reduce the risk when it was found. For example in Argentina, Leotta et al. [60], demonstrated the usefulness of a simple checklist (list included five groups of variables: situation and conditions of the building, equipment and tools, handlers, raw materials and products for sale, and production flow) as a risk quantification technique to identify relevant facts at butcher shops that should be corrected to improve the microbiological quality of the meat. Although they could significantly reduce EHEC O157:H7 contamination after implementation of the improved actions, they were not enough to eliminate the contamination completely, so other interventions are needed to reduce mainly the presence of pathogens in beef.

Different physical and chemical decontamination procedures have been evaluated worldwide to reduce the prevalence of STEC in meat, such as: hot water, organic acids and/or their salts, electrolytically-generated hypochlorous acid (EGHA), high hydrostatic pressure (HHP), irradiation, among others [61,62]. However, except for some particular publications [63], most of the studies evaluating the efficacy of decontamination interventions were proved under controlled conditions in laboratories. Even though some compounds have been approved by the European Commission or the US Department of Agriculture and the Food Safety and Inspection Service, intervention strategies should be validated in each abattoir considering the specific processing conditions. Taking into account those previously published paper, Signorini et al. [64] evaluated the antimicrobial effect of nine intervention strategies in situ against STEC on beef carcasses at commercial abattoirs. They found that automated application of lactic acid and hot water can be used efficiently to decontaminate beef carcasses to reduce public health risks associated with STEC. Other studies also alerted for a possible increase of Stx production by STEC strains in presence of some food additives, as was demonstrated by using sodium citrate [65] or brazilin and carvacrol [66]. So, when testing additives or other treatments, it is recommended to consider alternative approaches if there are bacteria harboring phage encoding toxins that can be induced with the treatment.

Although STEC foodborne outbreaks were historically associated to meat products, this pattern has changed since a larger number of food products are now associated with serious outbreaks, mainly linked to ready-to-eat products, including fresh fruits and vegetables. It has been reported that chlorine and chlorinated compounds and organic acids may have a limited effect on the reduction of these bacteria on the surface of certain food matrices, such as vegetables. Also, the use of chemical disinfectants is suspected to be environmentally unsafe and some of them are harmful to humans. These are some of the reasons why some countries forbid chlorinated disinfectants usage and many studies are searching for alternative natural antimicrobials.

The antibacterial activity of roselle calyx (*Hibiscus sabdariffa*) extracts (water, ethanol, methanol, acetone, and ethyl acetate) was tested against different foodborne bacteria, including STEC, on several food matrices, such as: leafy greens-romaine lettuce, spinach, coriander [67]; jalapeño and serrano peppers [68]; and mangoes [69]; among other food products [70,71,72]. In general, roselle calyx extracts resulted in a 2- to 3-log reduction in the concentrations of STEC strains, with the methanol and acetone extracts being the ones that produced a greater reduction. As this proved evident, roselle calyx methanol and acetone extracts (free of solvents) could be used as an alternative approach for reducing or eliminating foodborne bacteria, such as EHEC O157 or other STEC on raw fruits and vegetables. García-Heredia et al. [66], also determined the antimicrobial effect of whole plant extracts, such as *Lippia graveolens* and *Haematoxylon brassiletto*, and their known antimicrobial active components carvacrol and brazilin. This study concluded that the isolated compounds demonstrated a higher bactericidal effect compared with the whole plant extract, highlighting that the potential use of this natural alternatives as food preservatives should always be validated in foods, because using the extracts or their purified compounds could be different due to the presence of other unidentified compounds present in the crude extract. This is important to emphasize because, sometimes, the response to plant extracts are not only compound-dependent, but also dose-dependent and strain-dependent.

In summary, studies in Latin America indicated, as was highlighted in other published papers [73], that intervention methods should be considered as complementary measures, together with good agricultural and manufacturing practices throughout production process. Also, proper handling and processing practices need to be promoted and implemented among producers and consumers.

## 6. Epidemiology of STEC in Cattle

Cattle have been recognized as the main reservoir of STEC strains. However, systematic reviews and meta-analyses of literature to investigate the STEC prevalence in cattle at the global level showed that distribution of serogroups and virulence genes significantly differ by geographic region [74]. Several studies carried out in South America have contributed to the characterization of the STEC strains circulating in the region and to increase understanding of their features that could be linked to regional incidence of human disease.

In Argentina, several studies have been conducted to estimate the occurrence of STEC in cattle feces, carcasses, and hides. Recently, Cap et al. [75], reported a STEC isolation rate of 5% from hides and 8% from carcasses of two slaughterhouses. The STEC isolates belonged to serotypes O103:H2, O113:H21, O130:H11, O171:H2, O178:H19, ONT:H7 and ONT:H21. Brusa et al. [76], also detected a low prevalence (6%) of non-O157 strains isolated from beef carcasses, cuts and trimmings in slaughterhouses, and identified O8:H19, O130:H11, O174:H21, O178:H19 and O185:H7 as the most prevalent serotypes. These last studies are in agreement with previous reports, which showed that non-O157, and particularly O130:H11 and O178:H19, are the frequent serotypes among STEC strains isolated from dairy cows, beef abattoirs and feedlot cattle in Argentina [77]. Because STEC O157:H7 remains the most important serotype associated with HUS in this region, several recent studies continue assessing the prevalence and characteristics of this serotype. In a recent review, Pianciola and Rivas [78], observed that the estimated prevalence of *E. coli* O157 in Argentine cattle is near the world’s average and below the prevalences estimated for many other places with lower HUS incidence. Importantly, this review article supports the idea that isolation rates and serotype prevalences of bovine STEC are insufficient to explain the high HUS incidence in Argentina.

Therefore, analysis of the pathogenic potential of the circulating cattle strains and especially of those STEC belonging to serotypes associated to clinical cases, is critical for a better understanding of the regional differences in the incidence and the severity of human diseases. Stx subtyping is of great value because some types and subtypes of Stxs have been epidemiologically associated with different clinical outcomes after STEC infection. Particularly, the Stx2a subtype has been linked to high STEC virulence (reviewed in [13]). Regarding O157:H7 isolates, it has been observed that strains harboring only the *stx2c* subtype predominate in human samples from countries with low incidence of HUS [78]. In an analysis of O157 strains from Argentina, Pianciola et al. [18], identified that 76% human isolates and 56% bovine isolates harbored the *stx2a*/*stx2c* genotype, and that only 17% of the bovine isoles harbored *stx2c* as the only gene encoding Stx. These findings are consistent with previous studies reporting the prevalence of the *stx2a*/*stx2c* genotype among O157 isolates from cattle and patients in this country [79,80]. Other molecular typing methodolgies have also revealed similarity between bovine and clinical O157 isolates. Analysis by either Single Nucleotide Polymorphisms (SNP) identified a high predominance of the hypervirulent clade 8, while Lineage-Specific Polymorphism assay-6 (LSPA-6), showed preponderance of lineage I/II, which is linked to severity of the infections [18,81,82]. The presence in cattle of O157 strains with features similar to those strains that cause disease in humans could contribute to the high incidence of HUS in Argentina. However, the role of hypervirulence in pathogenesis has not been fully established yet. Noteworthy, sequence analysis indicated that *q*-*stx* region of *stx2a* and *stx2c* phages integrated in two STEC O157 isolated from cattle were identical to two O157:H7 strains associated with the spinach outbreak in USA [83].

Molecular analysis also point out that some STEC non-O157 circulating in cattle have a high pathogenic potential. Studies on *stx2a* expression of strains belonging to the O145 serogroup showed similar *stx2a* levels between bovine and human isolates [84]. Remarkably, genome comparison of a Stx2a prophage of a bovine STEC O145 against sequence databases, indicated a high identity with some phages detected in clinical O157:H7 strains from other countries [85]. Subtyping of *stx* from strains belonging to clinical-relevant serotypes showed that a proportion of O26:H11 strains harbored the *stx2a* genotype [86] and that O113:H21 strains mainly presented *stx2a* alone or together with *stx2c* [87].

In Brazil, variability has been observed in isolation rates and reported serotypes of bovine STEC strains. Comparative analyses show that strains beloging to O22:H8, O22:H16, O113:H21, O116:H21, and other serotypes have been commonly isolated from cattle from different Brazilian regions [8,88,89,90]. In an analysis of 105 STEC strains isolated from 1562 *stx*-positive fecal samples of healthy cattle in Rio de Janeiro, Gonzales et al. [90], found that O157:H7 was the most prevalent serotype (12,4%), followed by O113:H21 (7%) and O8:H19 (6%). It is interesting to note that human STEC infections in Brazil have been linked mostly to diarrhea cases and to non-O157 strains [4]. From the analysis of *stx* subtying, the study observed a high prevalence of *stx2c* among bovine STECs and, particularly, the presence of *stx2c* as the sole gene encoding Stx in all STEC O157 [90]. The authors suggested that the genotype predominant in the O157:H7 strains from the animal reservoir could contribute, at least in part, to the low occurrence of HUS in Brazil.

## 7. New Treatments against STEC Infections (Antisera and Vaccines)

Irrespective of the importance of EHEC/STEC infections in humans, there is no available licensed vaccine or effective therapy against these pathogens. Most regional studies performed between 2016 and 2018 involved a previously novel chimeric protein developed by Mejías et al. [91,92], which comprises a monomer of Stx2B subunit fused to the N-terminus of a monomer of *Brucella* lumazine synthase (BLS; BLS-Stx2B). This chimera was developed to increase the immunogenicity of Stx2B and as such, the conformational epitopes in Stx2B are stabilized in this chimeric protein. Studies in mice showed that BLS-Stx2B chimera is an efficient immunogen because it induced high titers of Stx2 antibodies against Stx2, Stx2 variants and Stx1 [91,92].

Sacerdoti et al. [93], demonstrated that female rats immunized with BLS-Stx2B, prior to pregnancy, developed high titers of IgG antibodies against Stx2B and were totally protected from abortion after challenged with a sublethal dose of Stx2. Further, the study described the passive transfer of antibodies from dams to their offspring, because they found specific anti-Stx2B IgG antibodies in pups’ sera at weaning. In addition, these antibodies conferred total protection against a lethal dose of Stx2 to all pups that were breastfed by the immunized dams.

Later, Mejías et al. [94], proposed that camelid antibodies could be suitable for neutralizing the Stx2 activity, because they possess unique characteristics. In this study, llamas were immunized with BLS-Stx2B chimera and Stx2B-specific variable heavy camelid domains (VHHs) antibodies selected. They developed bivalent [(2vb27)2] and trivalent [(2vb27)2-SA] molecules, the last ones with affinity to human and mouse serum albumin. Subsequently, they tested the in vivo Stx2-neutralization activity and protective capacity by injecting mice with either a lethal dose of Stx2 or by infection with STEC. The study demonstrated that [(2vb27)2-SA] was more effective to protect in vivo against Stx2 toxicity than ([(2vb27)2]). In addition, [(2vb27)2-SA] completely protected mice against the lethal intragastric STEC infection.

In another study, Hiriart et al. [95], obtained equine neutralizing hyperimmune serum anti-Stx1 and anti-Stx2 using IMC-Stx1B and IMC-Stx2B chimeras (BLS-Stx1B and BLS-Stx2B). Then, the F(ab`)2 fragments were purified, and their effectivity corroborated using Vero cells viability assays. They showed that these fragments were safe, and the maximum dose used in mice was well tolerated, and when a single dose or repeated doses were assayed in mice and rabbits, there were no clinical or subclinical alterations associated with the treatment. In addition, mice were protected from Stx2 intoxication, even when F(ab`)2 fragments were administered 48 h after the toxin. In addition, a cross-reactivity study with human tissues showed no specific binding of antibodies to the tissues. As a next step, a phase I clinical study to define safety, tolerability and pharmacokinetics on 14 healthy adult volunteers between 18 and 55 years old is in progress. The results will be presented to the National Administration of Drugs, Foods and Medical Devices (ANMAT) for evaluation, to request authorization for a phase II study in pediatric patients, and if successful, these F(ab`)2 fragments could be commercially available by the year 2020.

Cattle being the main reservoir of STEC O157:H7, Martorelli et al. [96], studied the effect of vaccination with two (Intimin C280 [IntC280], EspB) or three STEC antigens (IntC280, EspB, BLS-Stx2B) to reduce STEC shedding. The antibodies produced in sera from vaccinated animals using both antigens combinations inhibited O157:H7 adherence to epithelial cells and neutralized red blood cell lysis. The IgG immune response elicited against Stx2 was specific but lower compared to that obtained in mice with the same BLS-Stx2B chimera. Further, these antibodies were able to neutralize the cytotoxicity produced by Stx2 on Vero cells, even at 9 and 15 days after vaccination. Finally, the addition of BLS-Stx2B induced not only antibodies against Stx2, but also potentiated the IgG1 response to Intimin and EspB.

Next, the investigators showed that vaccination of calves with EspB + IntC280 or EspB + IntC280 + BLS-Stx2B, after challenged with STEC O157:H7, induced an increase of IntC280-, EspB-, Stx2B-specific antibodies in serum and intestinal mucosa. The Stx2B antibodies were efficiently transferred to the mucosa of the large and small intestine, as well as to the recto-anal junction. Both vaccines reduced O157:H7 shedding, but BLS-Stx2B addition did not confer further protection from shedding [97].

Navarro et al. [98], proposed other types of immunogens. They developed mimotopes of O157 lipopolysaccharide (LPS) by using phage display technology. These immunogens have the advantage to avoid lipid A endotoxicity. The authors selected the SP12 mimotope peptide because it was able to induce antibodies against O157 LPS and competed for binding sites in the same molecule. But so far, more studies are needed to support the use of this mimotope peptide as an alternative therapeutic immunogen.

Finally, nanoparticle formulations based on chemical-inactivated Outer Membrane Vesicles (OMVi) were obtained after detergent treatment of STEC O157:H7 and further glutaraldehyde inactivation. The OMVi were able to protect mice challenged with a concentrated culture supernatant of STEC O157:H7. Further, cattle showed a rapid decay in their humoral response after vaccination with OMVi. Overall, these studies suggested that OMVi-based formulations could be protective against STEC pathogenicity in mice and immunogenic in calves [99].

## 8. Discussion

As presented above, the Latin American groups have contributed significantly to our understanding of STEC/EHEC pathogenesis, deciphering the epidemiological distribution of serogroups in animals, food products and in different geographical locations. To further increase the collaboration of the STEC/EHEC investigators of Latin America with other groups working with this pathogroup and/or other pathogenic *E. coli* impacting humans and animals, the Latin American Coalition for *Escherichia coli* Research (LACER) was established in 2009 [100]. This collaborative group has aligned its mission to the One Health initiative, which integrates human medicine, veterinary medicine and environmental science to improve human and animal health, and as such the LACER group collaborates to understand *E. coli* disease and to find different therapeutic approaches for treatment and prevention of disease [100,101]. In the case of STEC/EHEC infections, the LACER group have facilitated collaborative efforts to diagnose, prevent and treat EHEC O157:H7 infections caused by consumption of contaminated food products that have resulted in the high prevalence of HUS cases observed in South America. This group is convinced that only way to address this endemic problem is by the collaboration of basic researchers, policy makers, physicians, veterinarians and other health professionals to find treatments and if required, develop affordable vaccines. As discussed above, LACER members are in the forefront in the development and evaluation of new experimental therapeutic approaches (e.g., complement inhibitors) and vaccines for the prevention and control of *E. coli* infections across pathogroups. The most notable advances are in novel EHEC O157:H7 vaccines for cattle and alternative treatments to combat Stx intoxication in humans.

Each section in this review is tailored to depict the work published by different Latin American groups in the past five years, without ignoring the seminal contributions by different groups worldwide. However, we decide to present it this way to highlight current regional advances but also to display challenges and health and public risk factors emerging in this region. For example, in the section describing STEC serotypes and novel virulence factors in isolates from humans and animals, we are bringing to the attention of the reader the prevalence and distribution of non-O157 STEC strains, the appearance of aEPEC strains with STEC genes as causative agents of human disease and the discovery of a novel pathogenicity island named LAA, that is mainly found in STEC LEE-negative strains. In the section discussing Shiga toxins and the immune system, we organized the information based on the organ affected and summarized a series of complications observed in human infections as a result of intoxication and the translational opportunities to develop a compound that target a specific mechanism blocking Shiga toxin. In the section about phages, studies of the basic biology of the *stx* phage are combined with anti-bacteriophage therapeutic approaches to combat the infection. Regarding the food products and food safety section, different interventions to decontaminate the food products and to eliminate bacterial contamination are discussed, with emphasis in the regional application of such methodologies. Further, the successful application of the intervention known as “safe butcher shops” is described. In the case of STEC epidemiology in cattle, this section presents the prevalence of serogroups and serotypes in Latin American cattle and describe the pathogenic potential of such isolates. Finally, the section describing new treatments against STEC infections emphasize the efforts to develop novel vaccine candidates that are suitable to be used in cattle and eventually humans, but also describes the development of other therapeutic approaches, such as an antisera against Stx that is currently being tested in phase I clinical trials.

**Figure 2:**
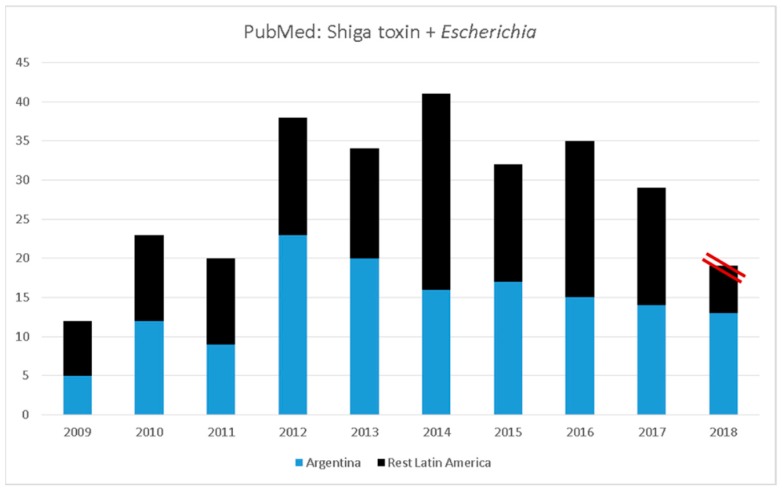
Summary of STEC/EHEC-related peer-reviewed publications by LACER members. A publication summary derived from the PubMed search engine based on numbers returned using the search terms “Shiga toxin” and “*Escherichia*” of the past 9 years, divided by authors from Argentina (light blue) and other Latin American countries (black). Red parallel lines indicated partial number of publications for 2018.

The result of LACER collaborative effort is evident in the increase of peer-review publications produced by Latin American investigators alone or in collaboration with groups in the region. Figure 2 shows PubMed results generated while searching Latin American countries (Argentina and other countries) and the words “Shiga toxin” and “*Escherichia*”. The results indicate that since the inception of the LACER group, a significant increase in the number of publications has occurred, with approximately half of the papers produced by Argentinian investigators. However, an important observation is that papers from other countries have significantly increase through the years and several manuscripts are now published by collaborations within regional groups.

## 9. Conclusions

Overall, Latin American investigators have made significant contributions to the advancement of STEC research, bringing several issues into perspective, including the importance of surveillance for new STEC isolates in the environment, the animal reservoir and eventually, those associated with human infections. Several studies have identified and characterized a wide variety of virulent genotypes of clinical relevance, maintaining specific attention to O157:H7, a serotype that remains causing human infections and a large proportion of HUS cases. Researchers have also been focusing in the molecular characterization of new virulence factors, the effects that the STEC toxins and the whole pathogen are having in the human host, but most importantly, developing novel immunogens, antibodies and vaccines, with the goal of protecting humans from STEC infections, but also reducing pathogen shedding from cattle. The significant advancement of these projects are placing these therapies closer to be tested in humans and animals and soon, commercialize to the public.

Integration of the scientific, public health, epidemiological and food safety knowledge has been possible thought the creation of networks, like the platform created by LACER [100,101]. However, further surveillance and epidemiology of EHEC/STEC infections and integration with global, regional and local Food Chain Surveillance Systems is required to ameliorate the damage STEC continues causing to human health in this part of the world.
